# Utility of 6% hydroxyethyl starch 130/0.4 in oral cancer surgeries with a duration of over 6 hours: A retrospective case-control study

**DOI:** 10.1097/MD.0000000000032958

**Published:** 2023-02-17

**Authors:** Erika Yaguchi, Tomoaki Ujita, Shinsuke Hamaguchi

**Affiliations:** a Department of Oral and Maxillofacial Surgery, Dokkyo Medical University School of Medicine, Mibu, Tochigi, Japan; b Department of Anesthesiology and Pain Medicine, Dokkyo Medical University School of Medicine, Mibu, Tochigi, Japan.

**Keywords:** circulatory maintenance, dilutional metabolic acidosis, hydroxyethyl starch, intraoperative blood loss, oral cancer surgery, renal dysfunction

## Abstract

To evaluate the utility of 6% hydroxyethyl starch (HES) 130/0.4 in oral cancer surgeries with durations over 6 hours. Using a case-control study design, the investigators enrolled patients who underwent oral cancer surgery involving osteotomy or manipulation near the major blood vessels at the Department of Orofacial Surgery in our hospital between 2017 and 2020. The predictor variable was 6% HES130/0.4. Outcomes included in-out balance and other postoperative parameters pertaining to circulatory maintenance (blood loss, urine volume, infusion volume, blood transfusion volume, albumin dose, hemoglobin levels, blood albumin levels, and doses of vasopressors used to maintain blood pressure), as well as pre- and postoperative renal function, pH, bicarbonate levels, and base excess. Changes in renal function were evaluated by assessing blood urea nitrogen and creatinine levels before surgery and at 1 and 7 days postoperatively. The Mann–Whitney *U* test was used for between-group comparisons, and Student *t* test was used for intragroup comparisons. The statistical significance was set at *P* < .05. A total of 65 patients underwent oral cancer surgery with a duration over 6 hours during the study period. The administration of 6% HES130/0.4 at 22.1 ± 7.5 mL/kg/day did not increase blood loss or the blood transfusion volume. Moreover, patients who were administered 6% HES130/0.4 had a significantly larger mean urine volume and infusion volume than those who were not administered 6% HES130/0.4. The infusion therapy could maintain the urine volume and did not worsen renal function. The results of this study showed that administration of 6% HES130/0.4 at a dose lower than 25 mL/kg in patients undergoing oral cancer surgery over 6 hours was effective for circulation maintenance but did not increase the intraoperative blood loss or transfusion volume. This treatment did not cause any dilutional metabolic acidosis or renal dysfunction.

## 1. Introduction

Red blood cell transfusion is sometimes required for head and neck surgeries, such as orthognathic and oral cancer surgeries, which are performed in regions near the major blood vessels.^[1]^ A previous retrospective study of 271 patients, who underwent bilateral sagittal split osteotomy or LeFort I osteotomy, reported a mean estimated blood loss of 345.2 ± 149.74 mL and a mean hemoglobin (Hb) level decrease of 2.38 ± 0.89 g/dL.^[2]^ It is recommended that additional management techniques should be used to avoid the need for blood transfusion in orthognathic surgeries for jaw deformity, particularly in young, healthy patients (American Society of Anesthesiologists physical status [ASA-PS] classification system I).^[3]^ Many patients, who undergo oral cancer surgery, are middle-aged or older adults with multiple comorbidities and have an ASA-PS score of II or higher. Furthermore, interventions, such as preoperative chemotherapy and radiation therapy, in these age groups greatly increase the risk of anemia, hypoalbuminemia, and renal dysfunction. Middle-aged and older adults are therefore highly likely to require blood transfusions during surgery. Nevertheless, adequate fluid management (e.g., with colloid solutions) should be ensured, to minimize the amount of blood transfusion required.^[4]^ This would lower the risk of blood transfusion complications, such as infection, transfusion-related acute lung injury, ABO- or non-ABO-related hemolytic transfusion reactions, febrile nonhemolytic transfusion reactions, transfusion-associated graft-versus-host disease, circulatory overload, and anaphylactic reaction.^[5]^

Colloid solutions and human albumin have been increasingly used in recent years for volume replacement in cases of perioperative blood loss and dehydration during surgery. Hydroxyethyl starch (HES), a plasma expander, is frequently used as a plasma substitute for volume therapy during long surgeries for oral and maxillofacial cancers. HES70/0.5 (Salinhes® or Hespander®; Fresenius Kabi, Bad Homburg, Germany) is an HES solution that has a long track record of use in Japan. However, in recent years, 6% HES130/0.4 (Voluven®; Fresenius Kabi, Bad Homburg, Germany), which is a tetrastarch, has become the most frequently used plasma expander in Japan.^[6]^ Nevertheless, to date, there have been no detailed investigations on whether 6% HES130/0.4 is suitable for use in circulatory maintenance or volume replacement in patients undergoing oral cancer surgery. The ability of 6% HES130/0.4 to minimize the amount of blood products used remains unknown. Furthermore, previous reports have suggested that 6% HES130/0.4 may be associated with a risk of postoperative kidney damage.

Therefore, we hypothesized that the use of 6% HES130/0.4 would be effective for circulatory maintenance, while minimizing the amount of blood products used, and would not increase the risk of postoperative kidney damage in patients undergoing surgery for oral cancer. The primary aim of this retrospective study was to examine whether administration of 6% HES130/0.4 was useful for circulatory maintenance in a cohort of patients who required blood transfusion during an oral cancer surgery that lasted over 6 hours. The secondary aim was to investigate the adverse effects on postoperative renal function and reductions in the use of blood products.

## 2. Methods

### 2.1. Patient selection

The study protocol was approved by Dokkyo Medical University Hospital’s Ethics Committee (approval number: *R*-24-15J) and all participants signed an informed consent agreement. This study was conducted in accordance with the guidelines of the 1964 Declaration of Helsinki.

This case-control study included patients who underwent oral cancer surgeries involving osteotomy or manipulation near the major blood vessels at the Department of Orofacial Surgery in our hospital, between 2017 and 2020. The inclusion criteria were as follows: Provision of written informed consent; ASA-PS score of II or below; Oral surgery duration > 6 hours; Normal range (or mild decrease) of preoperative renal function (i.e., absence of severe renal failure with an estimated glomerular filtration rate of ≥ 40 and no requirement for dialysis); Postoperative hospital stay of at least 1 week; No receipt of HES70 alone or a combination of 6% HES130/0.4 and HES70; No intraoperative pH correction with a sodium bicarbonate preparation; and No hemorrhagic diathesis.

The patients were divided into 2 groups based on whether they received patient group administered 6% hydroxyethyl starch 130/0.4 (H130 group) or did not receive 6% HES130/0.4 (control group). Those in the control group were administered only extracellular fluids, such as Ringer’s acetate or lactate solution.

### 2.2. Outcomes

The following variables were compared between the 2 groups: age; sex; height; body weight; preoperative Hb, hematocrit, blood albumin, pH, and bicarbonate ion (HCO_3_^-^) levels; base excess (BE); and surgery duration. The outcomes included the in-out balance and other postoperative parameters pertaining to circulatory maintenance (blood loss, urine volume, infusion volume, blood transfusion volume, albumin dose, Hb levels, blood albumin levels, and the dose of vasopressors [ephedrine/phenylephrine] used to maintain blood pressure), as well as pre- and postoperative renal function, pH, HCO_3_^-^ levels, and BE. Changes in renal function were evaluated by assessing the blood urea nitrogen (BUN) and creatinine (Cr) levels preoperatively and at 1 and 7 days postoperatively.

### 2.3. Statistical analysis

We used Altman nomogram^[7]^ and G*Power 3^[8]^ (University of Dusseldorf, Dusseldorf, Germany) to calculate the sample size. Our analysis revealed that 20 patients would be required to detect a significant difference regarding the utility of HES130/0.4 for circulatory maintenance, minimizing the amount of blood products used, and would not increase the risk of postoperative kidney damage in patients undergoing oral cancer surgery in each cohort, with a power of 0.8 and *P =* .05. Regarding the hypothesis tests for the secondary outcome measures, we mainly regarded the results as subsidiary information.

Data are presented as means ± standard deviations. Data analysis was performed using IBM SPSS Statistics 21® (IBM Corp., Armonk, NY). The Kruskal–Wallis 1-way analysis of variance with post hoc test or the Mann–Whitney *U* test was used as a nonparametric test for comparisons between the 2 groups, and Student *t* test was used for intragroup comparisons. The Bonferroni method was used for repeated-measures analysis of variance. A *P* value of < .05 was considered statistically significant.

## 3. Results

**Figure 1.**
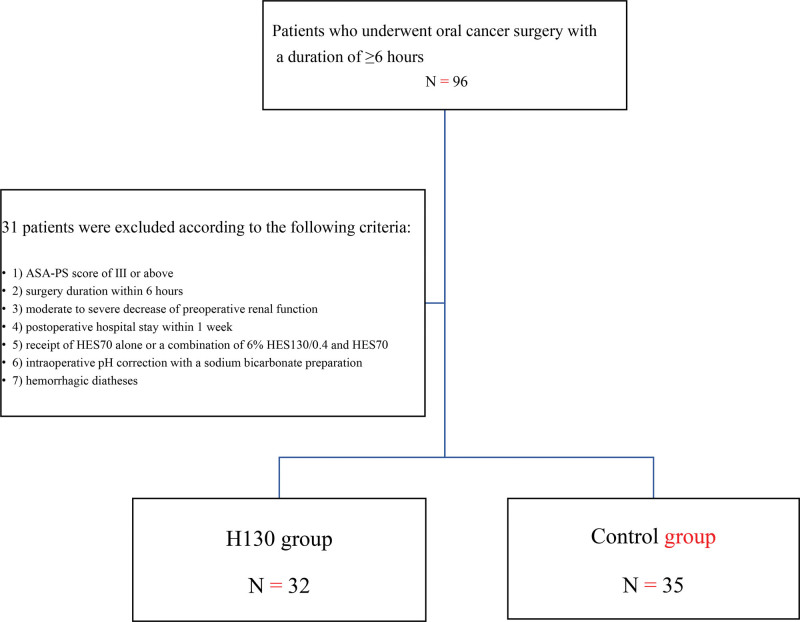
Process of patient selection. ASA-PS = American Society of Anesthesiologists physical status classification system, H130 = 6% hydroxyethyl starch 130/0.4, HES = hydroxyethyl starch.

A total of 96 patients underwent an oral cancer surgery with a duration > 6 hours at our hospital, between 2017 and 2020. Thirty-one patients did not meet the inclusion criteria. The remaining patients were categorized into either the H130 group (n = 32) or the control group (n = 35) (Fig. 1).

### 3.1. Patient backgrounds

Patient background characteristics are shown in Table 1. Both groups underwent general anesthesia with oxygen-air-sevoflurane (1.5–2.5%) inhalation and continuous administration of remifentanil (0.2–0.4 μg/kg/minutes). Patients in the H130 group were administered 6% HES130/0.4 with a mean (± standard deviation) dose of 1244 ± 561.5 mL (range, 500–3000 mL); the dosage per body weight was 22.1 ± 7.5 mL/kg/day. Extracellular fluid without a colloid solution was administered to maintain intraoperative blood pressure among patients in the control group. There were no significant differences in sex, age, height, body weight, or surgery duration between the 2 groups. There were also no significant between-group differences in preoperative Hb, albumin, HCO_3_^-^, BE, BUN, or Cr levels. An additional Microsoft Excel (Microsoft Corp., Redmond, WA) file provides more data on the patients [see Supplemental Digital Content, http://links.lww.com/MD/I462].

**Table 1:** Patient demographic and clinical characteristics.

	H130 group		Control		*P* value
(N = 32)		(N = 33)	
Mean	±SD	Mean	±SD
Patient characteristics					
Sex: M/F	M25/F7		M18/F15		
Age (yr)	61.9	±13.7	68.5	±14.6	.29
Height (cm)	163.6	±7.64	158.8	±9.55	.26
Weight (kg)	59.9	±15	54	±11.3	.27
Preoperative laboratory values					
Hemoglobin (g/dL)	11.8	±1.8	10.8	±1.9	.08
Hematocrit (%)	35	±6.18	34.4	±8.3	.88
Albumin (mg/mL)	3.98	±0.46	3.71	±0.54	.22
pH	7.4	±0.04	7.4	±0.06	.98
Bicarbonate ion (mEq/L)	24.3	±1.97	24.6	±1.97	.65
Base excess (mEq/L)	-0.15	±2.31	0.42	±2.04	.30
Blood urea nitrogen (mg/dL)	14.4	±4	17.4	±9.3	.08
Creatinine (mg/dL)	0.77	±0.17	1.31	±1.9	.11
Other data					
Surgical duration (h)	12.27	±0.15	11.01	±0.18	.07

F = female, H130 = 6% hydroxyethyl starch 130/0.4, M = male, SD = standard deviation.

### 3.2. Comparisons of in-out balance and postoperative parameters pertaining to circulatory maintenance

Values for in-out balance and various postoperative parameters pertaining to circulatory maintenance are shown in Table 2. There were no significant between-group differences in the amount of blood loss, in-out balance, blood transfusion volume, albumin dose, postoperative Hb levels, or blood albumin levels. The H130 group had a significantly larger mean urine volume than the control group (2511.9 ± 1664.5 mL vs 1828.7 ± 1301.7 mL; *P =* .029). The mean infusion volume was also significantly greater in the H130 group than in the control group (6997.1 ± 2687.3 mL vs 5331.3 ± 2471.1 mL; *P* = .012). In terms of vasopressor use, the amount of ephedrine or phenylephrine administered did not significantly differ between the 2 groups.

**Table 2:** Comparisons of fluid, blood, and acid-base parameters between the groups.

	H130 group		Control		*P* value	Significant difference
(N = 32)		(N = 33)	
Mean	±SD	Mean	±SD
Fluid management						
Blood loss (mL)	845.8	±842.3	477.2	±388.7	.05	
Urine volume (mL)	2511.9	±1664.5	1828.7	±1301.7	.03	†
Infusion volume (mL)	6997.1	±2687.3	5331.3	±2471.1	.01	†
In-out balance (mL)	3639.3	±1595	3025.4	±1478.6	.11	
Fluid transfusion						
Blood transfusion (mL)	306.3	±356.7	148.5	±291.1	.10	
Dose of albumin administration (mL)	140.6	±268.9	121.2	±380.8	.18	
Postoperative laboratory values						
Hemoglobin (g/dL)	9.5	±1.04	9.71	±9.7	.21	
Hematocrit (%)	29.4	±3.75	29	±6.3	.53	
Albumin (mg/mL)	3.31	±0.6	3.28	±0.51	.34	
Acid-base balance						
pH	7.4	±0.05	7.4	±0.04	.92	
Bicarbonate ion (mEq/L)	24.5	±1.76	25	±1.51	.21	
Base excess (mEq/L)	0.14	±2.07	0.71	±1.63	.42	
Administration of vasopressors						
Dose of ephedrine (mg)	19.4	±22.9	15.6	±20.3	.349	
Dose of phenylephrine (mg)	3.5	±3.1	5.2	±3.9	.235	

H130 = 6% hydroxyethyl starch 130/0.4, SD = standard deviation.

† indicates that there is significant difference between the groups.

### 3.3. Acid-base balance

There was no significant difference in the pH, mean HCO_3_^-^ level, or BE between the 2 groups (Table 2).

### 3.4. Assessment of perioperative renal function

**Figure 2.**
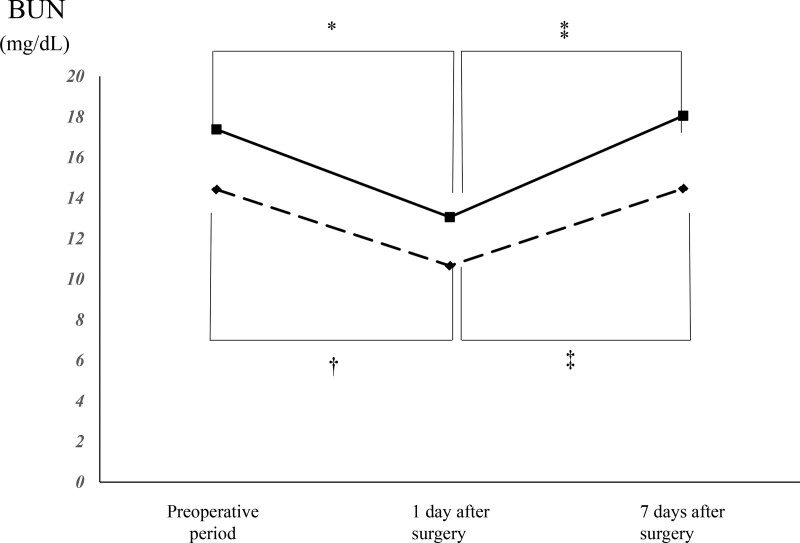
Perioperative change in the blood urea nitrogen levels within each group. There is a significant difference between two variables at each marked point. * versus the preoperative value in the control group. ⁑versus the value on postoperative day 1 in the control group. † versus the preoperative value in the H130 group. ‡ versus the value on postoperative day 1 in the H130 group. BUN = blood urea nitrogen, H130 = 6% hydroxyethyl starch 130/0.4.

**Figure 3.**
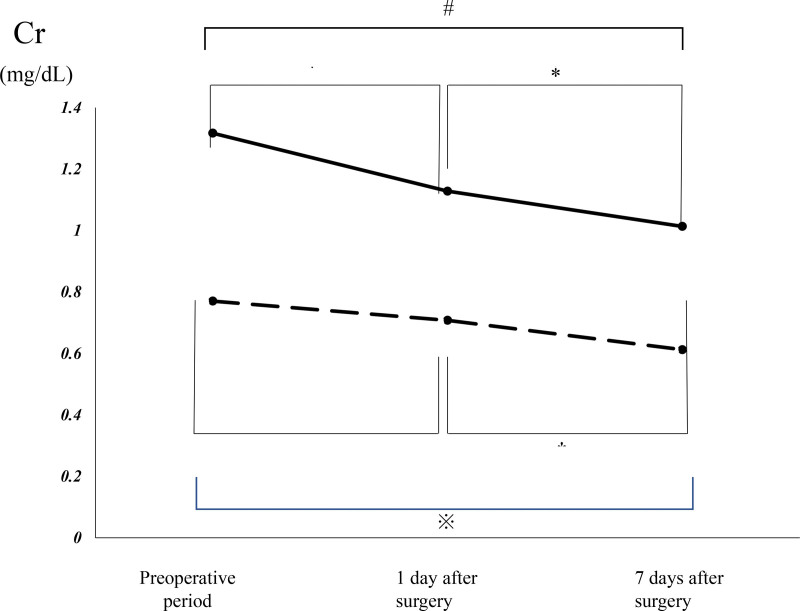
Perioperative change in the creatinine levels within each group. There is a significant difference between two variables at each marked point. * versus the preoperative value in the control group. ⁑ versus the value on postoperative day 1 in the control group. # versus the preoperative value in the control group. † versus the preoperative value in the H130 group. ‡ versus the value on postoperative day 1 in the H130 group. ※ versus the preoperative value in the H130 group. Cr = creatinine, H130 = 6% hydroxyethyl starch 130/0.4.

Table 3 shows the BUN and Cr levels of the 2 groups before surgery and on postoperative days 1 and 7. Figure 2 presents intragroup changes in mean BUN levels over time within groups. intragroup changes in the mean Cr levels over time within groups are presents in Figure 3.

**Table 3:** Changes in blood urea nitrogen and creatinine levels after surgery.

	H130 group		Control		*P* value	Significant difference
(N = 32)		(N = 33)	
Mean	±SD	Mean	±SD
Blood urea nitrogen (mg/dL)						
Preoperative value	14.4	±4	17.4	±9.3	.08	
1 d after surgery	10.7	±3.7	13.1	±7.7	.09	
7 d after surgery	14.5	±4.9	18.1	±8.2	.03	†
Creatinine (mg/dL)						
Preoperative value	0.77	±0.17	1.31	±1.9	.11	
1 d after surgery	0.71	±0.2	1.13	±1.47	.11	
7 d after surgery	0.61	±0.13	1.01	±1.13	.03	†

H130 = 6% hydroxyethyl starch 130/0.4, SD = standard deviation.

† indicates that there is significant difference between the groups.

Both groups exhibited a significant decrease in the BUN level on postoperative day 1 compared to the preoperative value of BUN (14.4 ± 4.0 mg/dL vs 10.7 ± 3.7 mg/dL, *P* < .001 in the H130 group; 17.4 ± 9.3 mg/dL vs 13.1 ± 7.7 mg/dL [*P* < .001] in the control group). Both groups subsequently exhibited a significant increase in the BUN level on postoperative day 7, as compared to postoperative day 1 (10.7 ± 3.7 mg/dL vs 14.5 ± 4.9 mg/dL [*P* < .001] in the H130 group; 13.1 ± 7.7 mg/dL vs 18.1 ± 8.2 mg/dL [*P* < .001] in the control group). However, there was no significant difference in the BUN level before surgery and on postoperative day 7 (*P* = .96 in the H130 group; *P* = .51 in the control group).

The Cr level significantly decreased from the preoperative assessment to postoperative day 1 (0.77 ± 0.17 mg/dL vs 0.71 ± 0.2 mg/dL [*P* < .001] in the H130 group; 1.32 ± 1.9 mg/dL vs 1.12 ± 1.47 mg/dL [*P* < .001] in the control group). Moreover, a significant decrease in the Cr level was also observed on postoperative days 1 and 7 in each group (0.71 ± 0.2 mg/dL vs 0.61 ± 0.13 mg/dL [*P* < .001] in the H130 group; 1.13 ± 1.47 mg/dL vs 1.01 ± 1.13 mg/dL [*P* = .03] in the control group). A significant decrease in the Cr level from the preoperative assessment to postoperative day 7 was observed in each group (0.77 ± 0.17 mg/dL vs 0.61 ± 0.13 mg/dL [*P* < .001] in the H130 group; 1.32 ± 1.9 mg/dL vs 1.01 ± 1.16 mg/dL [*P* = .024] in the control group).

Comparisons of the BUN and Cr levels between both the groups before surgery and on postoperative day 1 did not yield significant differences. There was also no significant difference in the Cr level between the groups on postoperative day 7. However, the H130 group had a significantly lower BUN level than the control group on postoperative day 7 (14.5 ± 4.9 mg/dL vs 18.1 ± 8.2 mg/dL, *P* = .03).

## 4. Discussion

The results of this retrospective observational case-control study indicated that the administration of 6% HES130/0.4 in oral cancer surgeries with durations > 6 hours did not increase the intraoperative blood loss or transfusion volume. Moreover, the administration of 6% HES130/0.4 was not associated with a risk of postoperative kidney damage. This conflicts with the results of prior studies.^[9,10]^ We found that the H130 group had a significantly greater intraoperative urine volume and infusion volume than the control group. There were no significant differences in pH, HCO_3_^-^, or BE between the groups, thus, indicating that 6% HES130/0.4 administration did not cause dilutional metabolic acidosis. The BUN levels in the H130 group were lower on postoperative day 1 compared to the preoperatively assessed BUN levels; preoperative BUN levels did not significantly differ from BUN levels on postoperative day 7. Cr levels also significantly decreased over time in both the groups. The H130 group had significantly lower BUN levels on postoperative day 7 than the control group.

Although 6% HES130/0.4 has a plasma-enhancing effect,^[11,12]^ it can also cause dilutional acidosis due to the absence of HCO_3_^-^ and dilutional anemia associated with the plasma-enhancing effect. According to the study by Kretschmer et al,^[1]^ a drop in the Hb levels during osteotomy procedures (e.g., bilateral orthognathic surgery) may be associated with an increase in surgery duration. Kasper et al^[10]^ reported significantly lower intraoperative Hb levels and a significant increase in the amount of blood products used in patients administered 6% HES130/0.4 compared to those in patients administered Ringer’s lactate solution or albumin. Skhirtladze et al^[13]^ also compared patients who were administered either 6% HES130/0.4 or albumin and Ringer’s lactate solution; while there was no significant difference in blood loss, the 6% HES130/0.4 group had a lower in-out balance, as well as a higher serum Cr level and amount of blood products used.

There is no definitive evidence that 6% HES130/0.4 is associated with increased blood loss, more frequent reoperation for bleeding, or greater blood product transfusion, or that these risks can be reduced by switching to a low-molecular-weight HES.^[14]^ As we limited our investigation to oral cancer surgeries that lasted > 6 hours, there was more blood loss than that reported by Salma et al^[2]^ While the H130 group tended to have more blood loss (845.8 ± 842.3 mL) than the control group (477.2 ± 388.7 mL), this did not reach statistical significance; furthermore, 6% HES130/0.4 administration did not lead to a significant decrease in the Hb or hematocrit levels and an increase of blood transfusion. We speculated that this result was correlated with the significantly higher urine volume in the H130 group compared with the control group in our study. The aforementioned discrepancy between the results of our study and those of Salma et al^[2]^ may be attributed to the lower 6% HES130/0.4 dosage range in the present study (21 mL/kg/day [1244 ± 561.5 mL] vs 50 mL/kg/day or less). Indeed, the dosage of 6% HES130/0.4 in the present study did not appear to have produced the agglutination-inhibiting effect that has been reported by a previous study.^[14]^ This may also account for the absence of dilutional acidosis that has been observed in other studies. Thus, our results indicate that a low dosage of 6% HES130/0.4 may decrease the risk of dilutional acidosis.^[15,16]^

In terms of hemodynamics, there was a trend for lower dosages of phenylephrine in the H130 group than in the control group; however, this did not reach statistical significance. Vasoconstrictor dosages may tend to be lower owing to the plasma volume expansion effects of 6% HES130/0.4.^[14]^

Many studies have shown that 6% HES130/0.4 is safe for renal function.^[17–21]^ Miyao and Kotake^[22]^ conducted a large retrospective cohort study to determine whether the use of 6% HES130/0.4 was related to kidney damage in patients undergoing surgery in Japan. They reported an acute kidney injury incidence of 6.2% among patients who received 6% HES130/0.4 and an incidence of 5.6% in the control group (odds ratio, 1.12; 95% confidence interval, 0.99–1.27; *P* = .07). This demonstrated that 6% HES130/0.4 does not increase the incidence or severity of postoperative acute kidney injury. In the present study, we used the BUN and Cr levels as indicators of renal function. Therefore, a significant decrease in the BUN level was observed from the preoperative period to postoperative day 1 in each group, and the BUN level increased from postoperative day 1 to postoperative day 7. However, the BUN levels on postoperative day 7 in each group were not significantly different than those preoperatively. Moreover, significantly lower Cr levels were observed over time in both groups. Particularly, the BUN and Cr levels in the H130 group on postoperative day 7 were significantly lower than those in the control group. According to these findings, we speculate that these results are related to the significantly greater urine volume in the H130 group than the control group; the plasma volume expansion effects of 6% HES130/0.4 maintained renal blood flow and it might have been ultimately reno-protective. This speculation is supported by the finding that the BUN and Cr levels on postoperative day 7 were not significantly different than the preoperative values.

Some limitations are acknowledged in the present study. First, there was no set standard for determining when blood transfusion was required. While red blood cell transfusion was generally performed when the Hb levels were ≤ 8 g/dL in men or ≤ 7 g/dL in women, in some instances the patient’s preoperative condition was the basis for performing blood transfusion. Second, we did not investigate the relationship between the amount of anesthesia administered during surgery and the duration of anesthesia. We also did not evaluate relationships among anesthesia-induced vasodilation, suppression of heart contraction force, and the doses of other vasopressors, such as dopamine, dobutamine, and noradrenaline. Third, the effects of respiration were not considered. Sodium bicarbonate preparations were not administered for pH correction in all cases. The intraoperative blood gas assessment did not evaluate whether there was a correction after changes were made in the respirator setting in response to a drop in pH (i.e., acidosis). While HCO_3_^-^ was added at the endpoints, there may have been potential secondary changes due to respiratory regulation. Finally, our follow-up period was limited to 7 days. While a longer follow-up period (e.g., 28 days) would have provided additional insights into the effects of 6% HES130/0.4, these data may have likely varied among patients due to postoperative treatments, such as chemotherapy and advanced surgery. Therefore, we decided to focus our analysis on the effects of intraoperative 6% HES130/0.4 administration up to postoperative day 7.

## 5. Conclusions

The results of this study indicate that the administration of 6% HES130/0.4 at a dose lower than 25 mL/kg in patients undergoing oral cancer surgery over 6 hours was effective for circulation maintenance, but did not increase intraoperative blood loss or transfusion volume. Furthermore, this infusion therapy may not worsen renal function due to increased urine volume and eliminated BUN.

## Acknowledgments

We would like to thank Dr Takashi Asai, a Professor in the Department of Anesthesiology of Dokkyo Medical University Hospital of Saitama, for providing helpful advices in statistical analyses of this study. Further, we would like to thank Editage (http://www.editage.jp) for English language editing.

## Author contributions

**Conceptualization:** Shinsuke Hamaguchi.

**Data curation:** Erika Yaguchi, Tomoaki Ujita.

**Supervision:** Shinsuke Hamaguchi.

**Writing – original draft:** Erika Yaguchi, Tomoaki Ujita, Shinsuke Hamaguchi.

**Writing – review & editing:** Shinsuke Hamaguchi.

## Supplementary Material
